# Heme, A Metabolic Sensor, Directly Regulates the Activity of the KDM4 Histone Demethylase Family and Their Interactions with Partner Proteins

**DOI:** 10.3390/cells9030773

**Published:** 2020-03-22

**Authors:** Purna Chaitanya Konduri, Tianyuan Wang, Narges Salamat, Li Zhang

**Affiliations:** Department of Biological Sciences, University of Texas at Dallas, Mail Stop RL11, 800 W. Campbell Road, Richardson, TX 75080, USA; pxk121430@utdallas.edu (P.C.K.); txw130830@utdallas.edu (T.W.); nxs158730@utdallas.edu (N.S.)

**Keywords:** Gis1, KDM4, heme, histone demethylase, protein interaction, JmjC domain

## Abstract

The KDM4 histone demethylase subfamily is constituted of yeast JmjC domain-containing proteins, such as Gis1, and human Gis1 orthologues, such as KDM4A/B/C. KDM4 proteins have important functions in regulating chromatin structure and gene expression in response to metabolic and nutritional stimuli. Heme acts as a versatile signaling molecule to regulate important cellular functions in diverse organisms ranging from bacteria to humans. Here, using purified KDM4 proteins containing the JmjN/C domain, we showed that heme stimulates the histone demethylase activity of the JmjN/C domains of KDM4A and Cas well as full-length Gis1. Furthermore, we found that the C-terminal regions of KDM4 proteins, like that of Gis1, can confer heme regulation when fused to an unrelated transcriptional activator. Interestingly, biochemical pull-down of Gis1-interacting proteins followed by mass spectrometry identified 147 unique proteins associated with Gis1 under heme-sufficient and/or heme-deficient conditions. These 147 proteins included a significant number of heterocyclic compound-binding proteins, Ubl-conjugated proteins, metabolic enzymes/proteins, and acetylated proteins. These results suggested that KDM4s interact with diverse cellular proteins to form a complex network to sense metabolic and nutritional conditions like heme levels and respond by altering their interactions with other proteins and functional activities, such as histone demethylation.

## 1. Introduction

**Figure 1:**
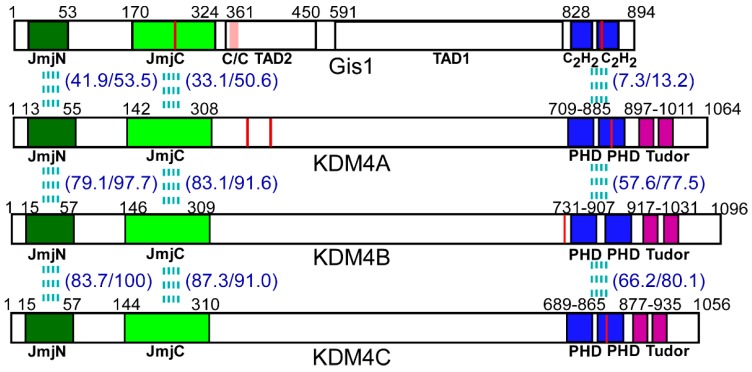
The structure and alignment of Gis1 and KDM4A/B/C domains. The domains of Gis1 and KDM4s are shown. Gis1 contains the JmjN + JmjC (JmjN/C) domain, the coiled-coil domain (C/C), two C2H2 type zinc fingers (ZnFs), and two transcription activation domains (TAD1 and TAD2). KDM4A/B/C proteins contain the JmjN/C domain, two PHD domains (each containing a conserved C4HC3 or C4HC2H motif), and two Tudor domains. The red lines represent cysteine-proline (CP) motifs, black lines indicate the start and end of the shown domains, and the pink box in the Gis1 structure denotes the coiled-coil domain. The % amino acid identity and similarity between Gis1 and KDM4s in JmjN/C and zinc finger domains are shown in parentheses.

Histone lysyl demethylases (KDMs) play important roles in chromatin remodeling and gene regulation by catalyzing the demethylation of methylated lysine residues on the N-terminal tails of histones [1]. The majority of KDMs are JmjC domain-containing proteins, which are dioxygenases that use α-ketoglutarate and Fe^2+^ to oxidize various substrates [2,3,4]. The KDM4 family, containing five members, i.e., KDM4A-E, accepts mono-, di- and trimethylated lysines as substrates [5,6]. KDM4A/B/C (Figure 1), but not KDM4D or E, contain the JmjC domain in the N-terminus as well as Tudor and plant homeodomain (PHD) domains in the C-terminus. KDM4A/B/C are widely expressed, and their orthologues are found among all vertebrates [7,8]. KDM4A/B/C share more than 50% sequence identity (Figure 1) and catalyze the demethylation of tri- and di-methylated forms of both histone H3 lysine 9 (H3K9me3/me2) and lysine 36 (H3K36me3/me2) [3,9,10,11,12,13]. KDM4 proteins play important roles in diverse biological processes, including oncogenesis, inflammation, and hematopoiesis [8,14,15,16]. It is worth noting that the histone demethylase activity of KDM4s requires α-ketoglutarate, O_2_, and Fe^2+^, which are important nutritional and metabolic indicators. Thus, histone demethylase activity is linked to and is highly responsive to nutrient status [17,18,19,20,21,22].

The yeast JmjC domain-containing protein Gis1 is an orthologue of KDM4 proteins. Previous data, including our own recent data, showed that Gis1 possesses histone demethylase activity at H3K36 [23,24,25,26]. In yeast, Gis1 is one of the key regulators mediating transcriptional reprogramming of carbon metabolism and stress responses during diauxic and post-diauxic shifts [27,28,29,30,31,32]. Gis1 binds to the post-diauxic shift (PDS) DNA element and can both activate and repress transcription [33,34,35]. It acts downstream of TOR, RAS/cAMP, and AKT/Sch9 signaling pathways in response to nutrient status [28,30,32]. Gis1 contains multiple domains, including a JmjN region, a JmjC region, a coiled-coil domain, two C2H2 type zinc fingers (ZnFs), and two transcription activation domains (TADs) (Figure 1) [30,36,37]. JmjN and JmjC interact physically to form a structural unit or a domain [37]. The JmjN/C domain presumably confers histone demethylase activity, but is dispensable for transcriptional activation by Gis1 [36]. This Gis1 JmjN/C domain is highly homologous to those of KDM4A/B/C, while the C-terminal regions of Gis1 and KDM4A/B/C share low homology (Figure 1). The Gis1 zinc fingers allow Gis1 to bind to DNA and regulate transcription [30,36,37]. The C-terminal regions of KDM4A/B/C proteins contain two PHDs and two Tudor domains (Figure 1). The PHD domain is a protein module containing ~50 amino acid residues with a conserved C4HC3 or C4HC2H motif that coordinates two zinc ions in a cross-brace configuration [38]. The Tudor domain typically contains ~60 amino acids that comprise 4–5 antiparallel β-strands to form a barrel-like structure [39]. Both PHD and Tudor domains are histone reader domains that recognize post-translationally modified histones, such as methylated H3 [40,41,42,43]. Although the JmjN/C domains of Gis1 and KDM4s are highly conserved, the C-terminal domain sequences of Gis1 are quite different, and there is no homology between the TADs of Gis1 and the corresponding regions of KDM4s (Figure 1). Thus, we do not expect that KDM4s can substitute for Gis1 in yeast.

Interestingly, our recent studies showed that heme promotes both transcriptional and histone demethylase activities of Gis1 [23]. Heme can bind to both the JmjN/C and ZnF domains, however, heme binding to the JmjN/C domain is not necessary for heme regulation of Gis1 activity. In contrast, heme binding to the ZnF domain promotes heme activation of both transcriptional and histone demethylase activities [23]. Under heme-deficient conditions, Gis1 is free of heme and is inactive. Its activities are repressed by the ZnF domain, which likely cooperates with other cellular proteins. When heme levels increase, heme binds to the JmjN/C and ZnF domains, presumably causing other cellular proteins to dissociate from Gis1 and alter Gis1 conformation, thereby leading to full activation of Gis1 transcriptional and demethylase activities. Heme acts as a prosthetic group or cofactor in proteins and enzymes required for oxygen utilization and metabolism, such as globins and cytochromes [44,45,46,47]. Heme serves as a signaling molecule regulating diverse processes ranging from gene transcription to circadian rhythm [48,49,50]. Heme directly binds to and modulates the activities of certain cellular proteins, such as the yeast heme-regulatory protein Hap1 [51,52] and the mammalian nuclear receptor Rev-erbα [49,53]. Experimental studies have increasingly shown that heme is a central signaling molecule in an array of physiological and pathological processes, including neurodegeneration, tumorigenesis, and adipogenesis [54,55,56].

Because Gis1 can sense heme and change its transcriptional and histone demethylase activities in response to heme, we asked if heme also regulates the activity of the human KDM4 family members, particularly, KDM4A/B/C which, like Gis1, not only contain the conserved JmjN/C domain, but also the C-terminal region with PHD zinc fingers (Figure 1). Interestingly, our data showed that heme strongly activates histone demethylase activity of KDM4A and C, but not KDM4B, while heme binds to all three. Furthermore, we identified other cellular proteins that are critical for heme regulation of Gis1. We found that 147 proteins could bind to Gis1 in vitro, notably, the transcriptional regulator Mot3 [57,58], which binds to Gis1 and is required for heme activation of Gis1 activity. Our data showed that heme is a conserved regulatory factor for KDM4 proteins and that heme regulation of Gis1 and likely other KDM4s involves a network of cellular proteins.

## 2. Materials and Methods

### 2.1. Yeast Strains and Plasmids

The yeast strains used were BY4741 (*MATa his3Δ1 leu2Δ0 met15Δ0 ura3Δ0*), MHY101 (*MATa ura3-52 leu2-3,112 his4-519 ade1-100 hem1-Δ100 URA3::PDS-lacZ*), MHY101Δgis1 (*MATa ura3-52 leu2-3,112 his4-519 ade1-100 hem1-Δ100 URA3::PDS-lacZ gis1::LEU2 ura3::Kan^r^*), MHY101*Δgis1Δmot3* (*MATa ura3-52 leu2-3,112 his4-519 ade1-100 hem1-Δ100 URA3::PDS-lacZ gis1::LEU2 mot3::HIS4 ura3::Kan^r^*), and BWG1-7b*Δgis1* (*MATa ura3-52leu2-3,112 his4-159 ade1-100 URA3::PDS-lacZ gis1::LEU2 ura3::Kan^r^*). The Yeast Tandem Affinity Purification (TAP) Fusion Library (scTAP) comprised of 4247 Open Reading Frames (ORFs), was purchased from Open Biosystems (Cat. No. YSC1177).

To delete the *GIS1* gene, MHY101 cells were transformed with a PCR product containing the *LEU2* gene in the middle and 44 bps sequences flanking the open reading frame sequence of *GIS1* on both sides. Knockout strains were confirmed by PCR and β-galactosidase assay. To delete the *MOT3* gene, MHY101*Δgis1* cells were transformed with a PCR product containing the *HIS4* gene in the middle and 44 bps sequences flanking the open reading frame sequence of *MOT3* on both sides. Knockout strains were confirmed by PCR. The PDS element-driven *LEU2-lacZ* reporter was introduced by transforming yeast strains with the with linearized, NcoI-cut pLS9-PDS plasmid, as described previously [34]. In the MHY101*Δgis1* strain, the *URA3* gene was deleted by transformation with a PCR product containing Kan^r^ sequence in the middle and 44 bps of sequence flanking the open reading frame sequence of *URA3* on both sides.

The PDS element-driven *LEU2-lacZ* reporter plasmid pLS9-PDS was as described [34], and was provided by Dr. Claudio de Virgilio’s lab (University of Fribourg, Fribourg, Switzerland). The pET-15b bacterial expression vector for expressing His_6_-tagged Gis1 from the T7 lac promoter was as described [59], and was provided by Dr. George M. Carman’s lab (Rutgers University, New Brunswick, NJ, USA). The pET-15b bacterial expression vector for expression of the His_6_-tagged JmjN/C domain of Gis1 from the T7 lac promoter was as described [23]. The JmjN/C domains of KDM4A/B/C were cloned into the T7 expression vector pET-15b cut with NdeI/BamHI, NdeI/XhoI, or XhoI/BamHI respectively. The coding sequences were confirmed by DNA sequencing (Eurofins MWG operon, Louisville, KY, USA). The yeast expression vectors for full-length Gis1 (pYY53), Gis1ΔJmjC (pYY54), and Gis1ΔZnF (pYY55) were as described [36], and were provided by Dr. Rolf Sternglanz’s lab (Stony Brook University, Stony Brook, NY, USA). The expression vector for Gis1ΔJmjN/C was as described [37,60], and was provided by Dr. Nianshu Zhang’s lab (University of Cambridge, UK). The expression vectors for the fusion proteins Hap1-GisΔZnF, Hap1-Gis1, Hap1-KDM4A, Hap1-KDM4B, and Hap1-KDM4C were constructed by inserting the coding sequences for the fusion proteins to the yeast expression vector SD5-HAP1 [61]. The DNA containing the coding sequences for fusion proteins was generated by overlapping PCR, as described previously [62]. The sequences of fusion proteins were confirmed by DNA sequencing using Eurofins MWG operon USA.

### 2.2. Cell Growth and β-Galactosidase Assays

Yeast cells were grown in rich yeast extract peptone dextrose (YPD) or synthetic complete media, as described previously [63,64]. Cell density was determined by measuring optical density at 600 nm. To determine β-galactosidase levels from reporter genes in Δ*hem1* cells bearing the PDS element-driven *LEU2-lacZ* reporter or the Hap1-driven UAS1-*CYC1-lacZ* reporter, cells were grown in synthetic complete medium containing a limiting amount of the heme precursor 5-aminolevulinic acid (ALA, 2.5 μg/mL) or a high amount of ALA (250 μg/mL). Cells were collected after they reached an optical density (600 nm) of approximately 1.0–1.5 to measure Gis1 and Hap1 activities. Collected cells were then subjected to chloroform permeabilization and β-galactosidase assays. The activities were measured and calculated in Miller units, as described previously [65,66].

### 2.3. Purification of KDM4 and Gis1 proteins, Spectroscopic Analyses, and Protein Binding to Heme–Agarose Beads

To purify the JmjN/C domains of KDM4A/B/C or full-length JmjN/C of Gis1 from *Escherichia coli*, BL21(DE3) bearing the pET-15b expression plasmids were grown to A_0.5_, then induced with 1 mM isopropyl β-d-1-thiogalactopyranoside (IPTG) for 2 h at 25 °C. Cells were collected and lysed with a French Press. The His_6_-tagged proteins were purified with Ni Sepharose 6 fast flow columns (GE Healthcare, Chicago, IL, USA), according to the manufacturer’s protocol. The eluted His_6_-Gis1 or His_6_-KDM4A/B/C proteins were desalted with PD-10 desalting columns (GE healthcare, Chicago, IL, USA), then cleaved with 0.005 U thrombin per μg of protein. Proteins without the tag were then purified from the mixture by passing them through the Ni Sepharose 6 fast flow columns again. To overcome nonspecific Ni binding, a stepped elution was performed with elution buffer containing increasing concentrations of imidazole (0, 40, 80, and 250 mM). All eluates were analyzed on SDS-PAGE gels.

Heme absorbance spectra were measured with a Varian Cary^®^ 50 UV-Vis Spectrophotometer. Samples contained 10 µM protein, 5 µM heme, and 0, 10, 20, 40, 80, or 160 mM imidazole. Protein and heme were prepared in 20 mM Tris (pH 8.0) and 500 mM NaCl; the imidazole stock was adjusted to pH 8.0 with HCl. Each sample was incubated for 30 s after the addition of heme prior to absorbance measurement.

To detect protein binding to heme with heme–agarose beads (Sigma, St. Louis, MO, USA), purified proteins were incubated with the beads in the heme binding buffer (20 mM Tris (pH 8.0), 500 mM NaCl, and 1% TritonX-100) for 1 h at 4 °C. After incubation, the beads were pelleted by centrifugation, and the supernatant was collected. The beads were then washed twice with heme-binding buffer. Subsequently, proteins bound to the beads and in the supernatant were electrophoresed on SDS polyacrylamide gels and visualized by Coomassie blue staining.

### 2.4. Measurement of KDM4A/B/C and Gis1 Demethylase Activity

The demethylase activity of purified KDM4A/B/C proteins was measured using an Epigenase Demethylase Activity/Inhibition Assay Kit (EpiGentek, P3081-48), according to the manufacturer’s protocol. Briefly, 50 ng of the trimethyl histone H3K9 peptide (Cat. No. R-1028), which was derived from residues within 1–100 of human histone H3, was stably coated onto 96-well microplate wells. Enzymatic reactions were set up for blank (without protein) and sample (with 300 ng, about 3 pmol, of purified KDM4A/B/C proteins) in a total volume of 50 μL, in the presence or absence of 2 μM heme. The microplate strip wells were covered with adhesive covering film and incubated at 37 °C for 120 min. To detect the amount of demethylated product, the wells were washed and incubated with a capture antibody (anti-H3K9 me1) at room temperature for 60 min followed by incubation with a detection antibody at room temperature for 30 min. The amount of demethylated product was fluorometrically measured with a BioTek Cytation 5-plate reader with excitation and emission wavelengths of 530 nm and 590 nm, respectively, after adding a fluorescence development solution. Demethylase activity of the protein was calculated in Relative Fluorescence Units (RFU) /min/mg, as described in the manufacturer’s protocol.

The demethylase activity of purified Gis1 proteins was measured using a custom-made Epigenase Demethylase Activity/Inhibition Assay Kit (EpiGentek, P3081-48 CUSTOM) with dimethyl histone H3K36 peptide (Cat. No. R-1038) anti-H3K36me1 capture antibody, as described above.

### 2.5. Identification of Gis1 Interactors, Mass Spectrometry (MS), and Proteomic Data Analysis

Yeast cell extracts were prepared according to previously established protocols [67,68]. Briefly, yeast cells were grown to an optical density (OD) of 1.0–1.5 in rich medium containing glucose under normoxic, hypoxic, intermediate, and high-heme conditions. The hypoxic (~10 ppb O_2_) growth condition was created using a hypoxia chamber (Coy Laboratory, Inc.) and it with a mixture of 5% H_2_ and 95% N_2_ in the presence of a palladium catalyst [69]. Intermediate and high heme conditions were achieved by growing Δ*hem1* yeast cells in the presence of intermediate (50 μg/mL) or high (250 μg/mL) levels of ALA. Cells were harvested and resuspended in three packed cell volumes of buffer (20 mM Tris, 10 mM MgCl_2_, 1 mM Ethylenediaminetetraacetic acid (EDTA), 10% glycerol, 1 mM dithiothreitol (DTT), 0.3 M NaCl, 1 mM phenylmethylsulfonyl fluoride, 1X protease inhibitors). Cells were then permeabilized by agitation with four packed cell volumes of glass beads and extracts were collected, as previously described [68].

His_6_-Gis1 was pre-bound to a Ni Sepharose 6 Fast Flow column (GE Healthcare, Chicago, IL). To identify the Gis1 interactors, the crude yeast protein extract was loaded onto a Gis1-bound column and incubated at 4 °C for 2 h. The complexes were then eluted from the column and separated by SDS-PAGE using 12% polyacrylamide gels followed by Coomassie blue staining. A total of 19 protein bands (see Supplemental Figure S2) were excised with sterile blades and destained twice with 100 mM NH_4_HCO_3_ in 50% acetonitrile. The samples were prepared following the Penn State Hershey Mass Spectrometry Core facility standard protocol. The Mass Spectrometry (MS) spectra were taken by ABSciex 5600 TripleTOF using samples separated by Eksigent NanoLC-Ultra-2D Plus and Eksigent cHiPLC Nanoflex via 200 µm × 0.5 mm chromatography. Multiple proteins were often identified in each band.

ProteinPilot software version 5.0 was used to perform protein identification by searching MS spectra against the species-specific (*Saccharomyces. cerevisiae*) National Center for Biotechnology Information (NCBI) database sequences, which contains 36,621 protein sequences plus 156 common lab contaminants. Bioinformatics analyses were performed using the paragon algorithm in ProteinPilot 5.0 software (Framingham, MA, USA) to identify the potential proteins interacting with Gis1 by ranking them according to their unused scores. Protein identifications must have a ProteinPilot Unused Score >1.3 (>95% confidence interval) in order to be accepted. In addition, the only protein IDs accepted must have a “Local False Discovery Rate” estimation of no higher than 5%, which is a more stringent criterion than the often-used 1% Global False Discovery Rate. The identified protein list was then uploaded to CRAPome to eliminate potential false positives [70]. Proteins with *p* values equal to 0 were selected and further analyzed. The total number of unique proteins identified was 148, including Gis1. The proteins were then grouped based on the conditions in which they were identified into various classes (high ALA only, intermediate ALA only, hypoxia only, air only, and a combination of these classes). Further Gene Ontology (GO) analysis of the identified proteins was performed using STRING (http://string-db.org/). Human orthologues of the yeast proteins were identified using YeastMine (https://yeastmine.yeastgenome.org/).

### 2.6. Preparation of Yeast Extracts, TAP-Pulldown, and Western Blotting

Yeast cells expressing various TAP-tagged proteins and full-length Gis1 or Gis1 deletion proteins were grown in synthetic complete media to an optical density (600 nm) of approximately 1.0–1.5. Cells were harvested and extracts were prepared as described previously [71]. For TAP-pulldown, protein extracts were incubated with IgG-Sepharose beads to capture the target proteins. After this binding reaction, the beads were washed to remove unbound proteins, as described [72]. All steps were carried out in the cold room at 4 °C. Input and protein complexes bound to the beads were detected by Western blotting, where 75 μg of proteins from each treatment condition were electrophoresed on 10% SDS–Polyacrylamide gels, then transferred onto an Immuno-Blot PVDF Membrane (Bio-Rad, Hercules, CA, USA). The membranes were probed with antibodies, followed by detection with a chemiluminescent Western blotting kit (Roche Diagnostics, Mannheim, Germany). The signals were detected with a Carestream image station 4000MM Pro and quantitation was performed with the Carestream molecular imaging software version 5.0.5.30 (Carestream Health, Inc., Rochester, NY, USA).

## 3. Results

### 3.1. Heme Binds to the JmjN/C Domain of KDM4A/B/C and Regulates the Histone Demethylase Activity of KDM4A and C, but not KDM4B

**Figure 2:**
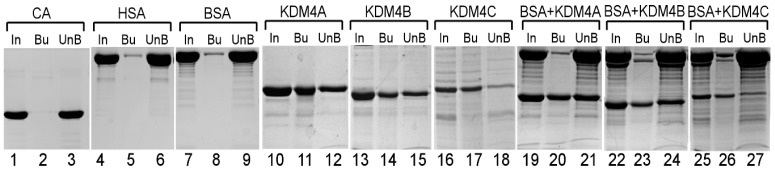
The pull-down of KDM4 JmjN/C domains by heme–agarose beads. Samples containing 500 pm of carbonic anhydrase (CA) (lanes 1–3), human serum albumin (HSA) (lanes 4–6), and bovine serum albumin (BSA) (lanes 7–9), and the JmjN/C domain of KDM4A (lanes 10–12), KDM4B (lanes 13–15), or KDM4C (lanes 16–18), or a mixture of the indicated proteins (500 pm each, lines 19–27) were incubated with heme–agarose beads. The input (In), bound (Bu), and unbound proteins (UnB) were analyzed by SDS-PAGE and are shown here.

**Figure 3:**
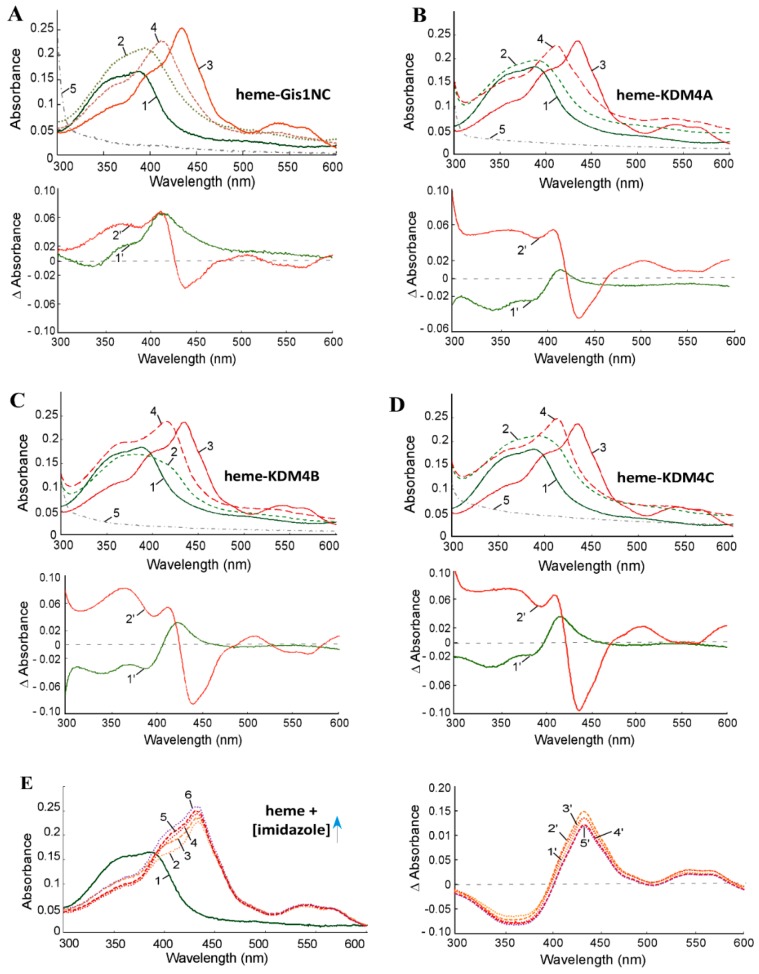
Absorption spectra of heme in the absence and presence of KDM4 JmjN/C domains. (**A**) Heme absorption spectra in the presence of the Gis1 JmjN/C domain (shown for comparison). (**B**) Heme absorption spectra in the presence of the KDM4A JmjN/C domain. (**C**) Heme absorption spectra in the presence of the KDM4B JmjN/C domain. (**D**) Heme absorption spectra in the presence of the KDM4C JmjN/C domain. (**E**) The effect of increasing concentrations of imidazole on the heme absorption spectrum. (**A–D**) Line 1: 5 μM heme; line 2: 5 μM heme + 10 μM JmjN/C domain of Gis1 or KDM4A/B/C; line 3: 5 μM heme + 10 mM imidazole; line 4: 5 μM heme + 10 μM JmjN/C domain of Gis1 or KDM4A/B/C + 10 mM imidazole; line 5: 10 μM JmjN/C domain of Gis1 or KDM4A/B/C. (**E**) Line 1: 5 μM heme; line 2: 5 μM heme + 10 mM imidazole; line 3: 5 μM heme + 20 mM imidazole; line 4: 5 μM heme + 40 mM imidazole; line 5: 5 μM heme + 80 mM imidazole; line 6: 5 μM heme + 160 mM imidazole. The difference spectra are shown under the heme absorption spectra. (**A–D**) Line 1′: (heme + protein) – heme; line 2′: (heme + imid + protein) – (heme + imid). E: Line 1′: (heme + 10 mM imidazole) – heme; line 2′: (heme + 20 mM imidazole) – heme; line 3′: (heme + 40 mM imidazole) – heme; line 4′: (heme + 80 mM imidazole) – heme; line 5′: (heme + 160 mM imidazole) – heme.

Previously, we showed that the purified JmjN/C domain of Gis1 binds to heme [23]. Here, we purified the JmjN/C domains (Figure 1) of KDM4A/B/C. We used previously well-established heme bead binding and heme spectrum methods [23,51,73] to determine if the JmjN/C domains of KDM4A/B/C bind to heme. First, using heme–agarose beads, we showed that the JmjN/C domains of KDM4A/B/C all bound to heme (Figure 2). For controls, we showed that carbonic anhydrase did not bind to heme agarose (lanes 1–3, Figure 2), as expected [74]. In contrast, both human serum albumin (has) (lanes 4–6, Figure 2) and bovine serum albumin (BSA) (lanes 7–9, Figure 2) bound to heme beads, because they bind to heme directly [74,75]. The JmjN/C domains of KDM4A/B/C all bound to heme beads strongly (lanes 10–18, Figure 2). When the JmjN/C domains of KDM4A/B/C were mixed in equal molar amounts with BSA and incubated with heme beads, they bound more strongly than BSA (lanes 19–24, Figure 2). Note that heme attached to the beads via the propionate group, thus, KDM4s do not interact with heme via the propionate group. Second, we examined heme binding to JmjN/C domains by detecting their effect on the major heme absorption band, the Soret band, which is around 400 nm. Like the JmjN/C domain of Gis1 (Figure 3A), JmjN/C domains of KDM4A/B/C (Figure 3B–D) bound to heme and shifted the Soret peak to longer wave lengths (Figure 3A–D, compare lines 1 and 2). The JmjN/C domains of Gis1 and KDM4A/B/C appeared to be purified without heme (Figure 3A–D), while full-length Gis1, which has higher heme-binding affinity than the JmjN/C domain, appeared to be purified with some heme [23]. As shown previously [51], imidazole chelated heme and shifted the Soret peak from about 386 nm to about 434 nm, and it also enhanced the spectral changes caused by the binding of the JmjN/C domain of Gis1 or KDM4A/B/C (compare lines 3 and 4 in Figure 3A–D). Specifically, in the presence of 10 mM imidazole, binding of the JmjN/C domains shifted the heme absorption peak from around 434 nm to 411 nm (compare lines 3 and 4 in Figure 3A–D). Figure 3E shows that 10–160 mM imidazole induced very similar heme spectral changes. Using imidazole titration as described previously [23], we found that similar concentrations of imidazole were required to shift the Soret peak to 434 nm in the presence of JmjN/C domains of KDM4-C compared to that of Gis1 (Supplemental Figure S1). The difference spectra shown in Figure 3A–D further illustrate the change caused by the binding of the JmjN/C domains to heme, particularly in the presence of imidazole, suggesting that the JmjN/C domains of KDM4A/B/C bound to heme with comparable affinity to the Gis1 JmjN/C domain.

**Figure 4:**
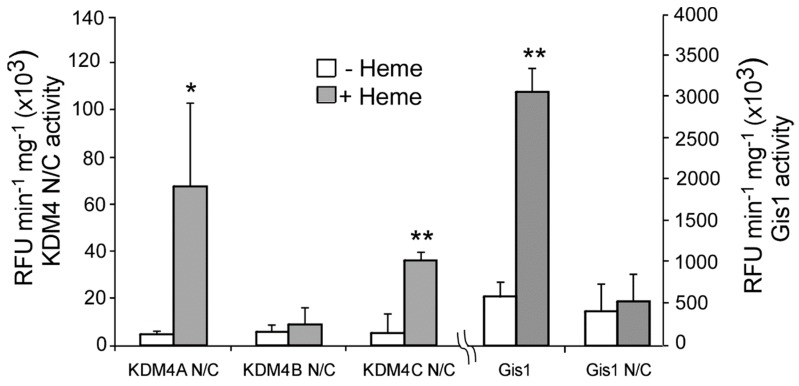
Heme stimulates histone demethylase activities of KDM4A and C, but not KDM4B. The H3K9me3 demethylase activities of purified KDM4 JmjN/C domains were measured as described in Section 2. The assays were repeated multiple times. The data plotted here are averages from at least three replicates. For statistical analysis, the activity in the presence of heme was compared to the activity in the absence of heme with a Welch 2-sample t-test. * *p* value < 0.05; ** *p* value < 0.005. The demethylase activities of full-length Gis1 and the Gis1 N/C domain are shown for comparison.

To determine whether heme binding to KDM4 JmjN/C domains affects their functions, we detected the effect of heme on histone demethylase activity using the well-established Epigenase Demethylase Activity/Inhibition Assay Kit (EpiGentek, Farmingdale, NY, USA). We detected the effect of heme on the activity of purified JmjN/C domains of KDM4A/B/C on H3K9me3. We found that heme strongly potentiated the activity of KDM4A and C, but not KDM4B (Figure 4). For comparison, we showed that heme stimulated the demethylase activity of full-length Gis1, but not the JmjN/C domain, as reported previously [23]. Clearly, the JmjN/C domains of KDM4A and C can mediate heme regulation of their histone demethylase activity, whereas those of KDM4B and Gis1 cannot.

### 3.2. The C-terminal Regions of KDM4A/B/C, like that of Gis1, Have the Potential to Modulate the Activity of the JmjN/C Domain and Heme Regulation

**Figure 5:**
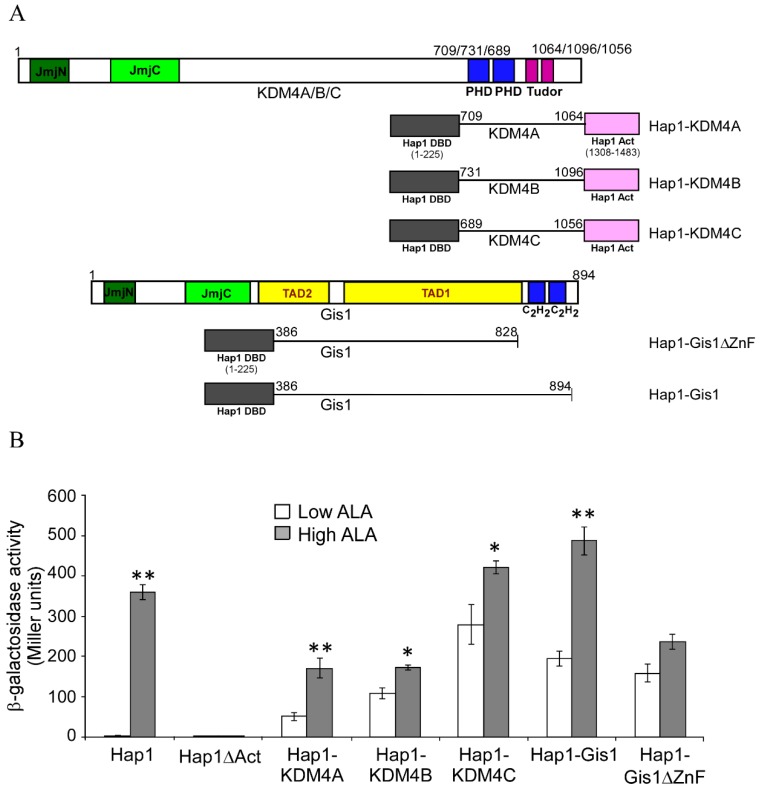
The C-terminal domains of KDM4 proteins can confer heme regulation of transcriptional activity via an unrelated transcription factor. (**A**) The domain maps of the hybrid proteins are shown. (**B**) The transcriptional activities of hybrid proteins in yeast cells. Yeast *Δgis1Δhem1* cells bearing the expression vector for wild type Hap1, Hap1ΔAct lacking the activation domain, Hap1-KDM4A, Hap1-KDM4B, and Hap1-KDM4C fusion protein, as well as the Hap1-binding UAS1-*CYC1-lacZ* reporter, were grown in the presence of low (2.5 μg/mL) or high (250 μg/mL) levels of ALA. Cells were then collected and β-galactosidase activities were measured. For statistical analysis, the levels in heme-deficient cells were compared to the levels in heme-sufficient cells with a Welch 2-sample t-test. * *p* value < 0.05; ** *p* value <0.005. The activities of Hap1-Gis1 and Hap1-Gis1ΔZnF are shown for comparison.

Previously, we showed that the C-terminal region of Gis1 containing the zinc finger domain promoted heme regulation when inserted between the DNA-binding and activation domains of a heterologous transcriptional activator Hap1 [23], as shown in Figure 5A,B (see Gis1-Hap1). We also inserted the C-terminal regions containing the PHD and Tudor domains of KDM4A/B/C between the DNA-binding and activation domains of Hap1 (Figure 5A,B). We found that the C-terminal regions of KDM4A, like that of Gis1, strongly promoted heme activation of transcriptional activity, while those of KDM4B and C somewhat promoted heme activation of transcriptional activity (Figure 5B). These results suggested that the C-terminal regions of KDM4A, like that of Gis1, and to a lesser extent, KDM4B and C, have the potential to modulate the activity of the JmjN/C domains and heme regulation.

### 3.3. Mass Spectrometry (MS) Identified 147 Proteins that can Interact with Gis1 In Vitro

**Figure 6:**
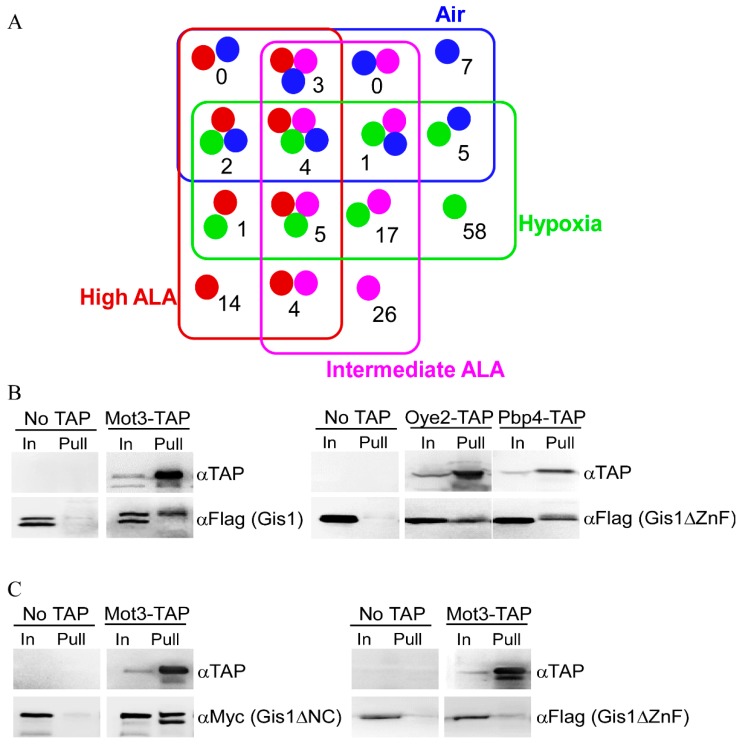
(**A**) Venn diagrams showing overlaps for Gis1-bound proteins under various conditions. The numbers of proteins bound to Gis1 identified from extracts prepared from cells grown under various conditions are shown here. (**B**) TAP pull-down confirms that Mot3, Oye2, and Pbp4 interact with Gis1. Extracts prepared from cells expressing TAP-tagged Mot3, Pbp4, or Oye2 and FLAG-tagged Gis1 were incubated with IgG-Sepharose 6 beads. Input proteins (In) and pulled-down (Pull) proteins were electrophoresed and detected by anti-TAP or anti-FLAG antibodies. Blots were probed with anti-TAP antibodies and then stripped and probed with anti-Flag antibodies. (**C**) The Gis1 ZnF domain, but not the JmjN/C domain, is required for interaction with Mot3. Extracts prepared from cells expressing TAP-tagged Mot3 and FLAG-tagged Gis1ΔZnF or Myc-tagged Gis1ΔNC were incubated with gG-Sepharose 6 beads. Input proteins (In) and pulled-down (Pull) proteins were electrophoresed and detected by anti-TAP, anti-Myc, or anti-FLAG antibodies. Blots were probed with anti-TAP antibodies and then stripped and probed with anti-Flag or anti-Myc antibodies.

Gis1, like other KDM4 proteins, is known to interact with many proteins [76]. These proteins may influence Gis1 histone demethylase and transcriptional activities and may promote heme regulation. Furthermore, Gis1 is likely to interact with different proteins under heme-deficient and heme-sufficient conditions. We therefore systematically identified the proteins that interact with Gis1 under heme-deficient and heme-sufficient conditions by coupling biochemical pull-down with mass spectrometry. To this end, we used yeast strains that were fully functional in oxygen and heme signaling [64]. We prepared extracts from *Δgis1* (to limit preformed Gis1 complexes) cells grown in air or under hypoxic conditions, and *Δgis1Δhem1* cells grown at high or intermediate heme levels. We used extracts prepared from hypoxic cells which could not synthesize heme [77] in lieu of heme-deficient cells because proteins are very labile in extracts prepared from heme-deficient cells. To identify Gis1-interacting proteins, extracts were loaded onto columns bound by full-length Gis1 purified from *E. coli*, and bound proteins were collected and subjected to SDS-PAGE. Protein bands that were retained specifically and only on Gis1-bound beads were excised (see bands shown in Supplemental Figure S2) and identified with mass spectrometry (MS). After extensive statistical and computational analyses (see Section 2), we identified 147 Gis1-interacting protein candidates (see Figure 6A). Several classes of the candidate proteins were worth noting, including 58 proteins that were identified only under hypoxic conditions that may suppress Gis1 activities in heme-deficient cells (see Table 1), 54 proteins that were identified only under high or intermediate levels of ALA or in air (heme-sufficient) that may promote Gis1 activities in heme-sufficient cells (see Table 2), and 36 proteins that were identified under both hypoxic/heme-deficient and heme-sufficient conditions that may both suppress and promote Gis1 activities depending on the heme levels (see Table 3). Seven of the identified Gis1-interacting proteins, Mig1, Snf1, Msc1, Bck1, Kcs1, Rvs167, and Cyr1 interact with Gis1 genetically [34,78,79,80,81], strongly supporting the validity of the biochemistry-MS approach used here. To further confirm the results from MS, we carried out co-immunoprecipitation for several identified Gis1-interacting protein candidates. Figure 6B shows that Mot3, Oye2, and PbP4 can be pulled down together specifically with Gis1, which further validates the results from the MS analysis. Interestingly, Figure 6C also shows that the ZnF domain of Gis1 is required for interaction with Mot3, while the JmjN/C domain is not necessary for interaction with Mot3. The lower band of the doublets shown in Figure 6B,C are presumably shorter, degraded fragments of the detected proteins lacking part of the N-terminal sequences (tags here are at the C-terminus).

Gene Ontology (GO) analysis of the identified Gis1-interacting proteins revealed several major, statistically significant functional classes, including 68 proteins, such as Gpd2 and Cpa1, that are able bind to heterocyclic compounds, such as nucleotides and nicotinamide adenine dinucleotide (Table 1, Table 2 and Table 3), 32 Gis1-interacting proteins that are involved in metabolism, such as the glycolytic pathway, the tricarboxylic acid cycle (TCA) cycle, and fatty acid metabolism (see Table 1, Table 2 and Table 3), 20 interacting proteins that are involved in ubiquitin-like protein (Ubl) conjugation (Table 1, Table 2 and Table 3), and 22 interacting proteins that are involved in acetylation (Table 1, Table 2 and Table 3). These results suggest that Gis1 interacts with proteins of many different pathways and potentially influences many molecular and cellular processes.

### 3.4. Mot3 is Essential for Heme Activation of Gis1 Transcriptional Activity

**Figure 7:**
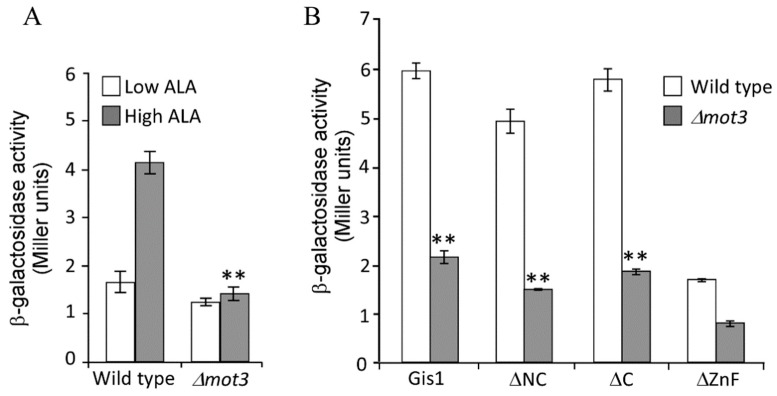
Mot3 is required for heme activation of Gis1 transcriptional activity in vivo. (**A**) Deletion of the *MOT3* gene reduces Gis1 transcriptional activity in heme-sufficient cells. Wild type *Δhem1* (WT) or *Δmot3Δhem1 (Δmot3)* cells bearing the PDS-*lacZ* reporter and expressing Gis1 were grown at a low (2.5 μg/mL) or high levels (250 μg/mL) of ALA. β-galactosidase activities (mean+SD) were measured from at least three independent cultures. For statistical analysis, the activities in wild type cells were compared to those in *Δmot3* cells at the same heme levels with a Welch 2-sample t-test. ** *p* value, 0.0001. (**B**) Deletion of the Gis1 JmjN/C domain does not abolish the requirement of Mot3 for heme activation. β-galactosidase activities (mean + SD) were measured from at least three independent cultures of yeast wild type (*HEM1* deletion) or *Δmot3* cells bearing the PDS-*lacZ* reporter and expressing Gis1 full-length or deletion proteins grown at high levels (250 μg/mL) of ALA. For statistical analysis, the activities in wild type cells were compared to those in *Δ*mot3 cells using a Welch 2-sample t-test. ** *p* value, 0.0001.

Mot3 is a known transcriptional regulator involved in the regulation of a subset of hypoxic genes [57,58,82,83]. Thus, we further ascertained its functional importance in heme regulation of Gis1 activity. Note that in wild type cells, which express both Gis1 and Mot3, Gis1 transcriptional activity was stimulated in heme-sufficient cells (high ALA, Figure 7A) relative to heme-deficient cells (low ALA). When the *MOT3* gene was deleted (*Δmot3*, Figure 7A), Gis1 transcriptional activity in heme-deficient cells remained at a similar level as in wild type cells. However, Gis1 activity in heme-sufficient cells was no longer higher than in heme-deficient cells in *Δmot3* cells. Figure 7A shows that deletion of *MOT3* did not affect Gis1 activity in heme-deficient cells, but abolished heme activation of Gis1 activity in heme-sufficient cells. Next, we examined the effect of *MOT3* deletion on the transcriptional activities of various Gis1 deletion mutants in heme-sufficient cells (cells grown in the presence of high levels of ALA, Figure 7B). We found that deletion of *MOT3* also strongly reduced the activity of Gis1 deletion proteins lacking JmjN and/or JmjC domains (Figure 7B). This result was consistent with previous studies showing that the JmjN/C domain does not affect Gis1 transcriptional activity [36]. Together, our results showed that Mot3 specifically interacts with Gis1 and is essential for heme activation of Gis1 transcriptional activity.

## 4. Discussion

The JmjC domain-containing proteins are highly conserved from yeast to humans, with 32 found in humans [8,11]. These proteins are dioxygenases and often possess histone demethylase activity [2,3,4]. The human JMJB2/KDM4 subfamily of histone demethylases has five functional members, namely, KDM4A–4E [8,11]. KDM4A, B, and C are broadly expressed in normal human tissues, while KDM4D and E (containing only JmjN+JmjC domain) are predominantly expressed in the testes in humans [13,84]. The functional domains of KDM4 proteins share high homology with Gis1 domains (Figure 1). KDM4 proteins, like Gis1, are responsive to nutritional and metabolic signals, in part due to their requirement for α-ketoglutarate (AKG), Fe^2+^, and O_2_ for activity [17,18,19,20,21]. KDM4 proteins play important roles in diverse biological processes, and their altered expression or function has been implicated in multiple cancers, cardiovascular diseases, and mental retardation [8,15,85,86,87,88].

Heme acts as a prosthetic group or cofactor in proteins and enzymes required for oxygen utilization and metabolism, such as globins and cytochromes [44,45,46,47]. Heme serves as a signaling molecule regulating diverse processes ranging from gene transcription to circadian rhythm [48,49,50]. Heme directly binds to and modulates the activities of certain cellular proteins, such as the yeast heme-regulatory protein Hap1 [51,52] and the mammalian nuclear receptor Rev-erbα [49,53]. In humans, heme deficiency causes serious diseases such as anemia and porphyria [89]. Conversely, high heme intake is associated with increased risk of cancer, type 2 diabetes, and coronary heart disease [90]. In yeast and mammals, intracellular heme levels are influenced by multiple factors including carbon sources, oxygen levels, heme availability, and circadian rhythm [77,89,91,92].

In this work, our data provide a direct link between KDM4 activities and heme, enabling the coordination of the regulation of histone demethylation, chromatin structure, and gene regulation with changes in heme levels and cellular stimuli and factors influencing heme levels. This link also provides an additional level of regulation of KDM4 activities by metabolic status relating to oxidative metabolism. The metabolic status influences KDM4 demethylase activity at two levels. The first level involves the requirement of α-ketoglutarate, Fe^2+^, and O_2_ for demethylase activity. α-ketoglutarate is a metabolite of the TCA cycle, which is a key part of oxidative metabolism. Interestingly, α-ketoglutarate is also a source of succinyl-CoA for heme synthesis [93]. Fe^2+^ and O_2_ are important nutrients and metabolic substrates which are required for heme synthesis. Heme synthesis also requires succinyl CoA, which is a metabolite of the TCA cycle. The second level of regulation is directly mediated by heme regulation of KDM4 histone demethylase activity. These two levels of regulation provide a tight regulatory scheme to coordinate KDM4 histone demethylase activity with the status of oxidative metabolism, oxygen, iron, and heme. Interestingly, although the JmjN/C domains of Gis1 and KDM4A/B/C are highly homologous (Figure 1) and can all bind to heme (Figure 2 and Figure 3), only those of KDM4A and C can mediate heme regulation of histone demethylase activity (Figure 4). Further structure–function analyses of these domains may shed light on the molecular codes underpinning heme regulation of histone demethylase activity.

The KDM4 proteins, particularly KDM4A/B/C and Gis1, contain multiple functional domains that are known to be versatile in their interactions with other macromolecules. A total of 195 protein–protein interactions (physical and genetic) were identified for Gis1, with 149 unique interactions (see https://thebiogrid.org/32151/summary/saccharomyces-cerevisiae/gis1.html). These interactions can be mediated by the JmjN/C domain, the C-terminal ZnF domain, or any other domains (Figure 1). PHD and Tudor domains of KDM4A/B/C are histone reader domains that interact with post-translationally modified histones, as well as other proteins and DNA [40,41,42,43]. These domains, like the JmjN/C domain, have high potential to interact with many protein partners. Indeed, most of the Gis1-interacting proteins have human orthologues (see Table 1, Table 2 and Table 3). These human orthologues are likely to interact with KDM4A/B/C proteins and influence diverse processes and pathways.

Interestingly, GO analysis of the Gis1-interacting proteins identified several prominent classes (Table 1: hypoxic conditions; Table 2: heme-sufficient conditions; Table 3: hypoxic and heme-sufficient conditions). The first class includes those proteins that bind to heterocyclic compounds, including 25 from hypoxic conditions, 19 from heme-sufficient conditions, and 24 from hypoxic and heme-sufficient conditions. Heterocyclic compounds are effective inhibitors of KDM4 activity [94,95], thus, it is fitting that many Gis1-interacting proteins can interact with heterocyclic compounds. The second class include those involved in metabolism, including nine from hypoxic conditions, 13 from heme-sufficient conditions, and 10 from hypoxic and heme-sufficient conditions. Gis1 and KDM4 proteins require the metabolites α-ketoglutarate, Fe^2+^, and O_2_ for activity. Furthermore, heme levels are directly linked to metabolic and nutritional conditions. Gis1 and KDM4 can sense heme levels and metabolic changes. The interactions of proteins involved in metabolism with Gis1 and perhaps KDM4 proteins are likely to facilitate the response of Gis1 and KDM4 proteins to changes in metabolic and nutritional conditions. The third class are those that can be conjugated by ubiquitin-like (Ubl) proteins, including seven from hypoxic conditions, four from heme-sufficient conditions, and nine from hypoxic and heme-sufficient conditions. Previous studies showed that ubiquitination and proteasomes are involved in controlling the levels of Gis1 and KDM4 proteins [60,96,97]. The association of Gis1 and likely KDM4 proteins with ubiquitination machinery probably allows them to associate with other ubiquitin-modified proteins. The fourth class include those that are acetylated, including twelve from hypoxic conditions, seven from heme-sufficient conditions, and three from hypoxic and heme-sufficient conditions. Methylation and acetylation are two important histone modifications that strongly influence chromatin structure and gene regulation. Previous studies indicated that crosstalk existed between acetylation and demethylation [98,99]. Our results in this work, which showed that many Gis1-interacting partners are acetylated, suggested that many potential crosstalk pathways exist between demethylation and acetylation.

It is also worth noting that C-terminal regions of KDM4 proteins, like the ZnF domain of Gis1, can promote heme regulation of unrelated transcriptional activators (Figure 5). This finding, alongside the data showing the effect of heme on histone demethylase activity of KDM4A and C, demonstrates that heme is a global regulator of KDM4 proteins. Furthermore, over 100 Gis1-interacting partners with diverse cellular functions exist, and these proteins often have human orthologues (Table 1, Table 2 and Table 3). Thus, the Gis1-KDM4 regulatory network appears to consist of many proteins and is well organized to respond to changes in metabolic and nutritional conditions. It is highly likely that human KDM4 proteins can interact with many proteins to influence diverse cellular pathways and processes.

## Figures and Tables

**Table 1:** List of bound proteins found only in extracts from hypoxic cells.

Common Name	ORF Name	Protein ID (gi #)	Description	Human Orthologues	HCB ^1^	Mtb ^2^	Ubl ^3^	Acet ^4^
ACT1	YFL039C	14318479	Structural protein involved in cell polarization	ACTA1, ACTA2, ACTB, ACTBL2, ACTC1, ACTG1, ACTG2, ACTR1A, ACTR1B, ACTRT1, ACTRT2, ACTRT3, POTEE, POTEKP	+		+	+
AHA1	YDR214W	398366155	Activator of heat-shock protein 90 ATPase	AHSA1				
AIM6	YDL237W	330443495	Altered inheritance of mitochondria protein	−				
ARC40	YBR234C	6319711	Subunit of the ARP2/3 complex	ARPC1A, ARPC1B				
BCK1	YJL095W	6322366	Serine/threonine protein kinase	MAP3K1, MAP3K2, MAP3K3, NRBP1, NRBP2, WNK1, WNK2, WNK3, WNK4	+			
CAF20	YOR276W	398366043	Cap-associated phosphoprotein of the mRNA cap-binding complex	-	+			
CDC33	YOL139C	6324433	mRNA cap binding protein and translation initiation factor	EIF4E, EIF4E2, EIF4E3	+		+	
CKS1	YBR135W	398364999	Cyclin-dependent protein kinase regulatory subunit and adaptor	CKS1B, CKS2				
CPR1	YDR155C	6320359	Cytoplasmic peptidyl-prolyl cis–trans isomerase	PPIA, PPIAL4E, PPIE, PPIF, RANBP2, RGPD1, RGPD3, RGPD4, RGPD5, RGPD6	+		+	+
CYR1	YJL005W	398364701	Adenylate cyclase	LRCH1, LRR1, LRRC59, LRRK1, PHLPP1, PHLPP2	+			+
DIG1	YPL049C	6325208	Downregulator of invasive growth	−	+			
DOT6	YER088C	398364577	Disruptor of telomeric silencing	−	+			
ERG6	YML008C	6323635	Delta(24)-sterol C-methyltransferase	COQ3, WBSCR27		+		+
ESS1	YJR017C	37362669	Peptidyl-prolyl cis–trans isomerase	PIN1				
GOR1	YNL274C	6325144	Glyoxylate reductase	GRHPR	+	+		
GPD2	YOL059W	6324513	Mitochondrial glycerol-3-phosphate dehydrogenase (NAD(+)) 2	GPD1	+	+		
GRX2	YDR513W	6320720	Mitochondrial glutaredoxin-2	GLRX, GLRX2				
GYP1	YOR070C	6324644	Cis-golgi GTPase-activating protein (GAP) for yeast Rabs	−				
HIT1	YJR055W	6322515	Protein involved in C/D snoRNP assembly	ZNHIT3				
HPA3	YEL066W	6320768	D-amino-acid N-acetyltransferase	−				+
HTB2	YBL002W	6319471	Core histone protein required for chromatin assembly and chromosome function	H2BFWT, HIST1H2BA, HIST1H2BB, HIST1H2BC, HIST1H2BD, HIST1H2BE, HIST1H2BF, HIST1H2BG, HIST1H2BH, HIST1H2BI, HIST1H2BJ, HIST1H2BK, HIST1H2BL, HIST1H2BM, HIST1H2BN, HIST1H2BO, HIST2H2BE, HIST3H2BB	+		+	+
IMP2	YMR035-W	398364225	Catalytic subunit of mitochondrial inner membrane peptidase complex	IMMP2L				
KCS1	YDR017C	398365025	Inositol hexakisphosphate and inositol heptakisphosphate kinase	IP6K1, IP6K2, IP6K3, IPMK	+	+		
MHP1	YJL042W	6322419	MAP-homologous protein	CARD8, CIITA, NLRC3, NLRC5, NLRP1, NLRP10, NLRP11, NLRP12, NLRP13, NLRP14, NLRP2, NLRP3, NLRP4, NLRP5, NLRP6, NLRP7, NLRP9, NLRX1, NOD1, NOD2			+	+
MIG1	YGL035C	6321403	Transcription factor involved in glucose repression	EGR1, EGR2, EGR3, EGR4, WT1	+			
MOT2	YER068W	398364501	General negative regulator of transcription subunit	CNOT4	+		+	
MRM1	YOR201C	398365713	Mitochondrial rRNA methyltransferase	−	+			
MRPL32	YCR003W	6319848	Mitochondrial ribosomal protein of the large subunit	MRPL32				
MRPS12	YNR036C	6324364	Mitochondrial ribosomal protein of the small subunit	MRPS12				
MSC3	YLR219W	6323248	Meiotic sister chromatid recombination protein	−				
NBA1	YOL070C	6324502	Protein of unknown function	−				
NUP60	YAR002W	6319318	Component of central core of the nuclear pore complex	−				
OSH3	YHR073W	6321864	Member of an oxysterol-binding protein family	OSBPL3, OSBPL6, OSBPL7				
OYE2	YHR179W	6321973	NADPH dehydrogenase	−	+			
PBP4	YDL053C	6320150	Pbp1p-binding protein	−				
PDI1	YCL043C	6319806	Protein disulfide isomerase	ERP27, ERP44, P4HB, PDIA2, PDIA3, TMX3				
PGA2	YNL149C	6324180	Essential protein required for maturation of Gas1p and Pho8p	−				+
PGI1	YBR196C	6319673	Glycolytic enzyme phosphoglucose isomerase	GPI		+		+
PGK1	YCR012W	10383781	3-phosphoglycerate kinase	PGK1, PGK2	+	+	+	+
PHO8	YDR481C	398366635	Repressible vacuolar alkaline phosphatase	ALPI, ALPL, ALPP, ALPPL2		+		
POL4	YCR014C	7839197	DNA polymerase IV	DNTT, POLL, POLM	+			
PSP2	YML017W	41629688	Asn-rich cytoplasmic protein containing RGG motifs	−	+			
PTC4	YBR125C	6319601	Cytoplasmic type 2C protein phosphatase (PP2C)	PPM1D, PPM1G				
RPS18A	YDR450W	6320658	Protein component of the small (40S) ribosomal subunit	RPS18	+			
SIP1	YDR422C	398366583	Alternate beta-subunit of the Snf1p kinase complex	−				
SNX41	YDR425W	6320633	Sorting nexin	SNX4				
SPC42	YKL042W	398364591	Central plaque component of spindle pole body	−				
SSD1	YDR293C	6320499	Translational repressor with a role in polar growth and wall integrity	DIS3L2	+			+
TMC1	YOR052C	6324626	AN1-type zinc finger protein, effector of proteotoxic stress response	−				+
TOD6	YBL054W	6319417	Twin of dot6p	−	+			
TOS1	YBR162C	6319638	Covalently bound cell wall protein of unknown function	−				
UTR1	YJR049C	6322509	ATP–NADH kinase	NADK	+	+		
YBL081W	YBL081W	6319390	Uncharacterized protein	−				
YCL020W	YCL020W	10383771	Retrotransposon TYA Gag gene co-transcribed with TYB Pol	−	+			
YDR365-WB	YDR365WB	7839164	Retrotransposon TYA Gag and TYB Pol genes	−				
YPK2	YMR104C	6323751	Serine/threonine protein kinase	AKT1, AKT2, AKT3, PRKCA, PRKCB, PRKCD, PRKCE, PRKCG, PRKCH, PRKCI, PRKCQ, PRKCZ, RPS6KB1, RPS6KB2, SGK1, SGK2, SGK3	+	+		
YPL247C	YPL247C	6325009	WD repeat-containing protein					
ZDS1	YMR273C	6323929	Protein with a role in regulating Swe1p-dependent polarized growth	−				

^1^ Heterocyclic compound binding; ^2^ metabolism; ^3^ Ubl conjugation; ^4^ acetylation.

**Table 2:** List of bound proteins found only in extracts from normoxic or heme-sufficient cells.

Common Name	ORF Name	Protein ID (gi #)	Description	Human Orthologues	HCB ^1^	Mtb ^2^	Ubl ^3^	Acet ^4^
ADE1	YAR015W	6319326	Phosphoribosylaminoimidazole-succinocarboxamide synthase	PAICS	+	+		+
ADH3	YMR083-W	6323729	Mitochondrial alcohol dehydrogenase isozyme III	SORD, TP53I3		+		
AGE1	YDR524C	398366675	ADP-ribosylation factor (ARF) GTPase activating protein (GAP) effector	ACAP1, ACAP2, AGAP1, AGAP2, ARAP1, ARAP2, ARAP3, ASAP1, ASAP2, ASAP3				
ALT1	YLR089C	6323118	Alanine transaminase (glutamic pyruvic transaminase)	CCBL1, CCBL2, GPT, GPT2	+	+		
ATG18	YFR021W	16740527	Autophagy-related protein	WIPI1, WIPI2				
AYR1	YIL124W	398364253	Bifunctional triacylglycerol lipase and 1-acyl DHAP reductase	DECR1, DHRS7, DHRS7B, DHRS7C, HSD11B1, HSD17B2				
BOI1	YBL085W	6319386	Protein implicated in polar growth	NEB, NEBL, PSD, PSD4, SH3RF2				
BRE2	YLR015W	6323043	Subunit of COMPASS (Set1C) complex	ASH2L	+			
BUD5	YCR038C	10383799	Bud site selection protein	RAPGEF1, SOS1, SOS2				
COA6	YMR244C-A	6323902	Protein involved in cytochrome c oxidase (Complex IV) assembly	COA6				
CPA1	YOR303W	398366173	Carbamoyl-phosphate synthase arginine-specific small chain	CAD, CPS1, OTC	+	+		
ERG26	YGL001C	6321437	Sterol-4-alpha-carboxylate 3-dehydrogenase	GMDS, HSD3B1, HSD3B2, HSD3B7, NSDHL		+		
ERV41	YML067C	6323573	Protein localized to COPII-coated vesicles	ERGIC2				
GIS2	YNL255C	6324074	Translational activator for mRNAs with internal ribosome entry sites	CNBP	+			
GVP36	YIL041W	398364435	BAR domain protein	TAF7, TAF7L, FECH			+	+
HEM15	YOR176W	398365579	Ferrochelatase	FECH		+		
HFD1	YMR110C	6323757	Fatty aldehyde dehydrogenase	ALDH3A1, ALDH3A2, ALDH3B1, ALDH3B2		+		
HSP42	YDR171W	6320376	Small heat-shock protein (sHSP) with chaperone activity	−				
INA22	YIR024C	6322215	Inner membrane assembly complex subunit 22	−				
IPP1	YBR011C	6319483	Cytoplasmic inorganic pyrophosphatase	PPA1, PPA2			+	
IRC22	YEL001C	6320836	Increased recombination centers protein	−				
IRC5	YFR038W	42742173	Uncharacterized ATP-dependent helicase	HELLS, SMARCAL1	+			
LYS12	YIL094C	6322097	Mitochondrial homoisocitrate dehydrogenase	IDH3A	+	+		
LYS20	YDL182W	6320019	Homocitrate synthase isozyme	HMGCL		+		
LYS21	YDL131W	6320071	Mitochondrial homocitrate synthase	HMGCL		+		
MIG3	YER028C	6320866	Transcription corepressor	EGR1, EGR2, EGR3, EGR4, WT1	+			
MLF3	YNL074C	398365051	Serine-rich protein of unknown function	−				
MPE1	YKL059C	6322791	Essential conserved subunit of CPF cleavage and polyadenylation factor	RBBP6	+			
MSC1	YML128C	6323507	Protein of unknown function	−				
NAM9	YNL137C	398364685	37S ribosomal protein	−	+			
NPA3	YJR072C	398365155	GPN-loop GTPase	GPN1	+		+	
OSM1	YJR051W	6322511	Fumarate reductase	SDHA				
PHB2	YGR231C	50593217	Prohibitin	PHB2				
PNO1	YOR145C	6324720	Pre-rRNA-processing protein	−	+			
PUB1	YNL016W	6324312	Nuclear and cytoplasmic polyadenylated RNA-binding protein	RBM42, TIA1	+			+
RNH1	YMR234W	6323890	Ribonuclease	RNASEH1				
RPC40	YPR110C	6325367	RNA polymerase subunit AC40	POLR1C	+			+
RRP3	YHR065C	37362659	ATP-dependent rRNA helicase	DDX28, DDX47	+			
RSA1	YPL193W	6325063	Protein involved in the assembly of 60S ribosomal subunits	NUFIP1				+
RTN1	YDR233C	398366209	Reticulon-like protein 1	RTN1, RTN2, RTN3, RTN4				
RVS167	YDR388W	6320596	Calmodulin-binding actin-associated protein	−			+	+
SCS2	YER120W	398364741	Integral ER membrane protein	MOSPD3, VAPA, VAPB				+
SDS22	YKL193C	6322655	Regulatory subunit of the type 1 protein phosphatase	CD180, CNTRL, DNAAF1, LRRC32, NRROS, PPP1R7, TLR2, TLR3, TLR4, TLR5, TLR7				
SHE1	YBL031W	6319440	Mitotic spindle protein	−				
SIP2	YGL208W	6321230	One of three beta subunits of the Snf1 kinase complex	PRKAB1, PRKAB2		+		
SNF4	YGL115W	6321323	Activating gamma subunit of the AMP-activated Snf1p kinase complex	PRKAG1, PRKAG2, PRKAG3	+	+		
SOK1	YDR006C	398364967	Protein of unknown function	TCP11				
SRB4	YER022W	6320860	Subunit of the RNA polymerase II mediator complex	MED17	+			
SSO1	YPL232W	6325024	Plasma membrane t-SNARE	STX11, STX12, STX17, STX1A, STX1B, STX2, STX3, STX4, STX7				
SUA7	YPR086W	6325343	Transcription factor TFIIB	GTF2B				
TDH1	YJL052W	398364523	Glyceraldehyde-3-phosphate dehydrogenase	GAPDH, GAPDHS	+	+		
VMA13	YPR036W	6325293	Subunit H of the V1 peripheral membrane domain of V-ATPase	ATP6V1H				
YBL086C	YBL086C	6319385	Uncharacterized protein	FAM102A				
YCR087C-A	YCR087C-A	6319930	Putative protein of unknown function	−	+			

^1^ Heterocyclic compound binding; ^2^ metabolism; ^3^ Ubl conjugation; ^4^ acetylation.

**Table 3:** List of bound proteins found in extracts from both hypoxic and normoxic or heme-sufficientcells.

Common Name	ORF Name	Protein ID (gi#)	Description	Human Orthologues	HCB ^1^	Mtb ^2^	Ubl ^3^	Acet ^4^
ATP1	YBL099W	330443397	Alpha subunit of the F1 sector of mitochondrial F1F0 ATP synthase	ATP5A1, ATP5B	+	+		
BEM2	YER155C	398364959	GTPase-activating protein	ARHGAP10, ARHGAP12, ARHGAP15, ARHGAP21, ARHGAP23, ARHGAP26, ARHGAP27, ARHGAP35, ARHGAP42, ARHGAP9, CHN1, CHN2, GMIP, HMHA1, OPHN1, RACGAP1	+		+	
DBP5	YOR046C	6324620	Cytoplasmic ATP-dependent RNA helicase of the DEAD-box family	DDX19B, DDX25	+			
DLD3	YEL071W	6320764	D-2-hydroxyglutarate--pyruvate transhydrogenase	AGPS, D2HGDH, LDHD	+	+	+	
DUF1	YOL087C	6324485	Uncharacterized WD repeat-containing protein	WDR48				
ERG11	YHR007C	6321795	Lanosterol 14-alpha-demethylase	CYP51A1	+	+	+	
GAL83	YER027C	6320865	One of three possible beta-subunits of the Snf1 kinase complex	PRKAB1, PRKAB2		+		
GUA1	YMR217W	6323873	GMP synthase	GMPS	+	+	+	
HBT1	YDL223C	6319978	Shmoo tip protein	−				
HRK1	YOR267C	6324841	Serine/threonine protein kinase	HUNK, PRKAA2	+			
ILV1	YER086W	6320930	Mitochondrial threonine dehydratase	−				
IST2	YBR086C	27808701	Increased sodium tolerance protein	ANO1, ANO2, ANO3, ANO4, ANO5, ANO6, ANO7				
MOT3	YMR070W	6323715	Transcriptional activator/repressor	SMARCA2, SMARCA4	+			+
NMA1	YLR328W	6323360	Nicotinic acid mononucleotide adenylyltransferase	NMNAT1, NMNAT2, NMNAT3	+	+		
NMA2	YGR010W	6321447	Nicotinic acid mononucleotide adenylyltransferase	NMNAT1, NMNAT2, NMNAT3	+	+		
NOG2	YNR053C	6324381	Nucleolar GTP-binding protein	GNL2	+			
OLE1	YGL055W	398364717	Acyl-CoA desaturase	SCD, SCD5	+	+		
OPY1	YBR129C	6319605	Protein of unknown function	PLEK, PLEK2				
PAL2	YHR097C	6321889	pH-response regulator protein	−				
PBP1	YGR178C	398366039	PAB1-binding protein	ATXN2, ATXN2L	+		+	
RPT1	YKL145W	6322704	ATPase of the 19S regulatory particle of the 26S proteasome	PSMC2	+			
RTK1	YDL025C	6320179	Probable serine/threonine protein kinase	HUNK, PRKAA2	+		+	
RVB2	YPL235W	6325021	RuvB-like protein	RUVBL2	+			
SNF1	YDR477W	398366631	AMP-activated S/T protein kinase	HUNK, MELK, PRKAA1, PRKAA2, STK40, TRIB1, TRIB2, TRIB3	+	+	+	
SOF1	YLL011W	6323018	Protein required for biogenesis of 40S (small) ribosomal subunit	DCAF13	+			
SRO9	YCL037C	37362625	Cytoplasmic RNA-binding protein	LARP1, LARP4B	+		+	
SRV2	YNL138W	6324191	CAP (cyclase-associated protein)	−				
TUB1	YML085C	6323554	Tubulin alpha-1 chain	TUBA8	+			
TUB2	YFL037W	14318481	Beta-tubulin, associates with alpha-tubulin	TUBB, TUBB1, TUBB2A, TUBB2B, TUBB3, TUBB4A, TUBB4B, TUBB6, TUBE1	+			
UGP1	YKL035W	398364619	UTP--glucose-1-phosphate uridylyltransferase	UAP1, UGP2		+		+
VTS1	YOR359W	398366369	Flap-structured DNA-binding and RNA-binding protein	SAMD4A	+			
WHI2	YOR043W	6324617	Growth regulation protein	−				
YBR238C	YBR238C	6319715	Mitochondrial membrane protein	−	+			
YCK2	YNL154C	6324175	Palmitoylated plasma membrane-bound casein kinase I (CK1) isoform	CSNK1G2	+		+	+
YGR237C	YGR237C	6321676	Uncharacterized protein	−				

^1^ Heterocyclic compound binding; ^2^ metabolism; ^3^ Ubl conjugation; ^4^ acetylation.

## Supplementary Materials

The following are available online at https://www.mdpi.com/2073-4409/9/3/773/s1, Figure S1: Absorption spectra of heme bound to the JmjN/C domain of Gis1 or KDM4A/B/C in the presence of increasing concentrations of imidazole; Figure S2: Proteins pulled down with His6-tagged Gis1 in the presence of yeast extracts prepared from cells grown under normoxic conditions (**A**), hypoxic conditions (**B**), intermediate amounts of heme precursor ALA (**C**), or high amounts of ALA (**D**).
