# Supplementary material for: Heme, A Metabolic Sensor, Directly Regulates the Activity of the KDM4 Histone Demethylase Family and Their Interactions with Partner Proteins
Source: Cells. 2020 Mar 22;9(3):773. doi: 10.3390/cells9030773 (PMC7140707; doi:10.3390/cells9030773)
Supplement: Supplementary file 1 [file cells-09-00773-s001.pdf]

SUPPLEMENTARY MATERIALS

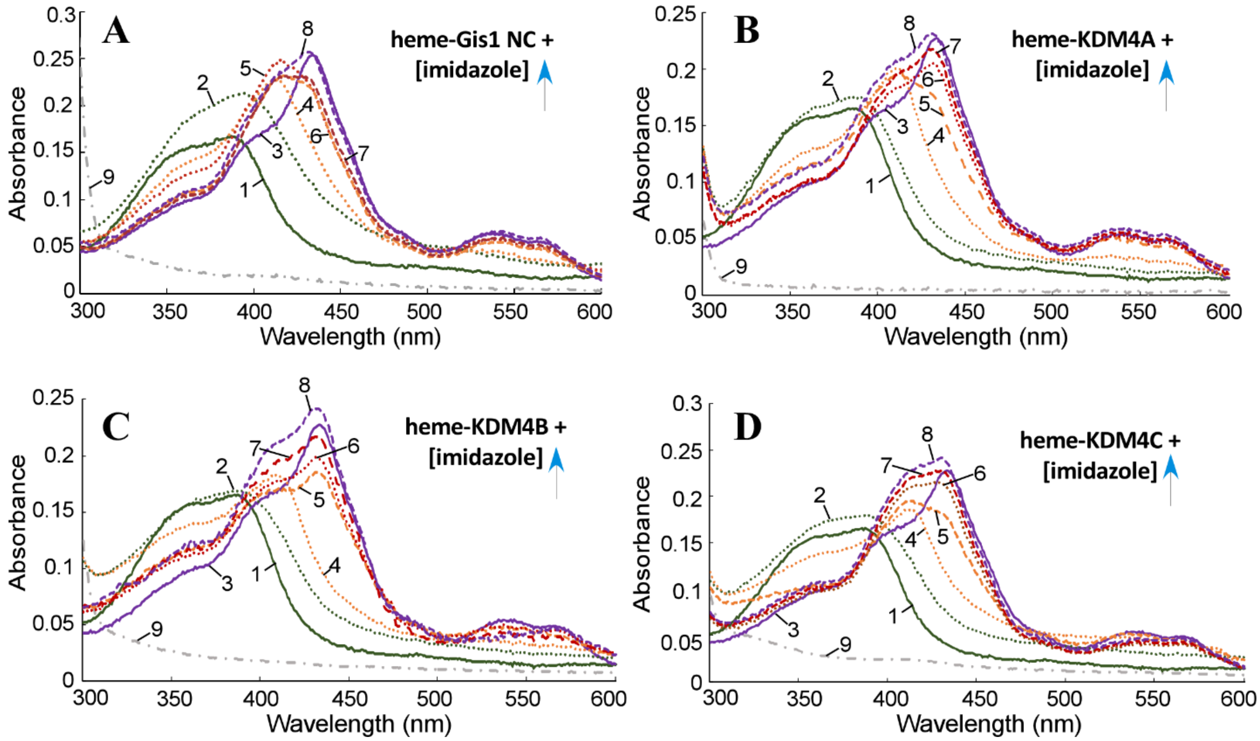

Supplemental Figure 1. Absorption spectra of heme bound to the JmjN/C domain of Gis1 or KDM4A/B/C in the presence of increasing concentrations of imidazole. (A) The shifting of heme- Gis1 JmjN/C domain absorption peak from about 406 nm to 434 nm by increasing concentrations of imidazole. (B) The shifting of heme-KDM4A JmjN/C domain absorption peak from about 406 nm to 434 nm by increasing concentrations of imidazole. (C) The shifting of heme-KDM4B JmjN/C domain absorption peak from about 406 nm to 434 nm by increasing concentrations of imidazole. (D) The shifting of heme-KDM4C JmjN/C domain absorption peak from about 406 nm to 434 nm by increasing concentrations of imidazole. line 1: 5 μM heme, line 2: 5 μM heme + 10 μM JmjN/C domain of Gis1 or KDM4A/B/C, line 3: 5 μM heme + 10 mM imidazole, line 4: 5 μM heme + 10 μM JmjN/C domain of Gis1 or KDM4A/B/C + 10 mM imidazole, line 5: 5 μM heme + 10 μM JmjN/C domain of Gis1 or KDM4A/B/C + 20 mM imidazole, line 6: 5 μM heme + 10 μM JmjN/C domain of Gis1 or KDM4A/B/C + 40 mM imidazole, line 7: 5 μM heme + 10 μM JmjN/C domain of Gis1 or KDM4A/B/C + 80 mM imidazole, line 8: 5 μM heme + 10 μM JmjN/C domain of Gis1 or KDM4A/B/C + 160 mM imidazole, line 9: 10 μM JmjN/C domain of Gis1 or KDM4A/B/C.

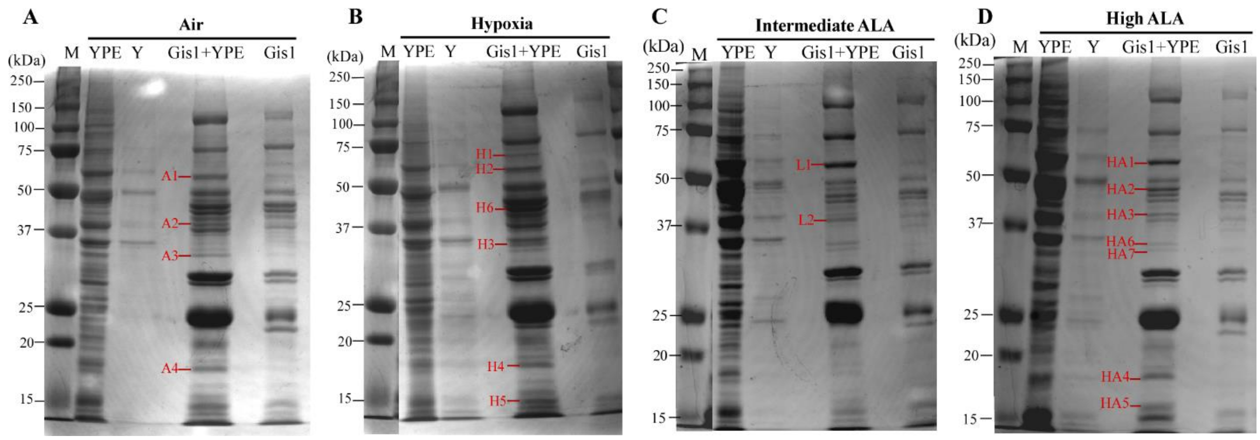

Supplemental Figure 2. Proteins pulled down with His6-tagged Gis1 in the presence of yeast extracts prepared from cells grown under normoxic (A), hypoxic (B), intermediate amounts of heme precursor ALA (C), or high amounts of ALA (D) conditions. Proteins pulled down from yeast extracts by His6-tagged Gis1 were analyzed by SDS-PAGE followed by Coomassie blue staining. Bands marked in red were excised, and proteins were detected by using mass spectrometry. M: Protein markers, YPE: Yeast protein extracts, Y: proteins bound to Ni Sepharose beads when incubated with yeast extracts, Gis1+YPE: proteins bound to Ni Sepharose beads that are bound with His6-Gis1 when incubated with yeast extracts, Gis1: purified His6-Gis1.
